# Facial tightening using a novel vacuum‐assisted microneedle fractional radiofrequency system: A prospective, randomized, split‐face study

**DOI:** 10.1111/jocd.16414

**Published:** 2024-06-24

**Authors:** Ying Liu, Wenxin Yu, Jiafang Zhu, Qingqing Cen, Lucia Zhou, Gang Ma, Ying Shang, Yue Han, Xiaoxi Lin

**Affiliations:** ^1^ Department of Laser and Aesthetic Medicine, Shanghai Ninth People’s Hospital, Shanghai Jiao Tong University School of Medicine Shanghai China; ^2^ Department of Plastic and Aesthetic Surgery, Nanfang Hospital Southern Medical University Guangzhou China; ^3^ Department of Plastic and Reconstructive Surgery, Shanghai Ninth People's Hospital Shanghai Jiao Tong University School of Medicine Shanghai China

**Keywords:** microneedle fractional radiofrequency, rejuvenation, skin laxity, vacuum‐assisted facial tightening, wrinkles

## Abstract

**Background:**

The microneedle fractional radiofrequency system (MFRS) is able to rejuvenate facial appearance by heating and coagulating certain depth of skin tissue.

**Objective:**

To evaluate the safety and efficacy of a novel vacuum‐assisted MFRS for facial contour tightening.

**Methods:**

This prospective, randomized, split‐face study included 21 patients who underwent three treatments with a vacuum‐assisted MFRS at 1‐month intervals. Half of the face was treated with the MFRS; the other half was untreated (control). Facial volume changes and wrinkles were objectively measured using a three‐dimensional imaging system and VISIA‐CR.

**Results:**

Volume changes of the treated midface were −0.24 ± 0.75, −0.59 ± 0.92, and −0.55 ± 0.65 mL at 1, 3, 6 months follow‐up; however, measurements of the control side were 0.08 ± 0.70, −0.08 ± 0.53, and − 0.10 ± 0.86 mL, indicating significant reductions (*p* < 0.05). The number of facial wrinkles on the treated side was significantly reduced to 12.44 ± 4.85 at 3 months and sustained at 6 months (11.11 ± 4.100) compared to the control side (14.89 ± 5.26 and 13.22 ± 4.44, respectively; *p* < 0.05). No long‐term side effects occurred.

**Conclusion:**

The vacuum‐assisted MFRS is safe and effective and is recommended for improving facial tightening and reducing wrinkles. This technology is sufficient to ensure the insertion depth, thus helping to improve the treatment accuracy and safety. The MFRS provides sustained effects for at least 6 months.

## 1 INTRODUCTION

Facial photoaging, which is characterized by facial wrinkles, sagging fat pad, and unsatisfactory contour, is a major concern for aging Asian subjects. Severe facial wrinkles can be improved by injection and filling treatments, whereas severe skin laxity can be improved by aesthetic surgery. However, individuals with mild or moderate facial skin laxity may not have indications of aesthetic surgery, and the majority of them may not accept the associated long downtime and scar formation. Less invasive therapy including focused ultrasound or radiofrequency, has been gradually introduced to meet the demands for mild skin laxity.^1^

The microneedle fractional radiofrequency system (MFRS) was approved in 2009, and its efficacy for treating facial laxity, reducing wrinkles, and providing other facial rejuvenation results has been widely confirmed.^2^ The MFRS can transform electromagnetic radiation into electricity. When the microneedle is inserted into biological tissues, the electric current causes the movement of charged particles and induces dermal heating and collagen coagulation.^3^ The thermal damaged tissue is able to induce collagen remodeling and skin contraction, thus leading to clinical improvement of mild to moderate wrinkles and skin laxity.^4^, ^5^

However, during MFRS treatment, facial areas without the support of bones, such as the cheek, may be difficult to treat if the skin is too loose for the operator to insert the tip of MFRS, and the insertion depth may be difficult to control precisely. Therefore, a novel vacuum‐assisted MFRS was introduced to solve these problems and provide a more precise and safe treatment (Figure 1). To our knowledge, this is the first prospective, single‐blind, split‐face study to investigate the clinical efficacy of the novel vacuum‐assisted MFRS for wrinkles and skin rejuvenation using objective indicators and visual evaluations.

**FIGURE 1:**
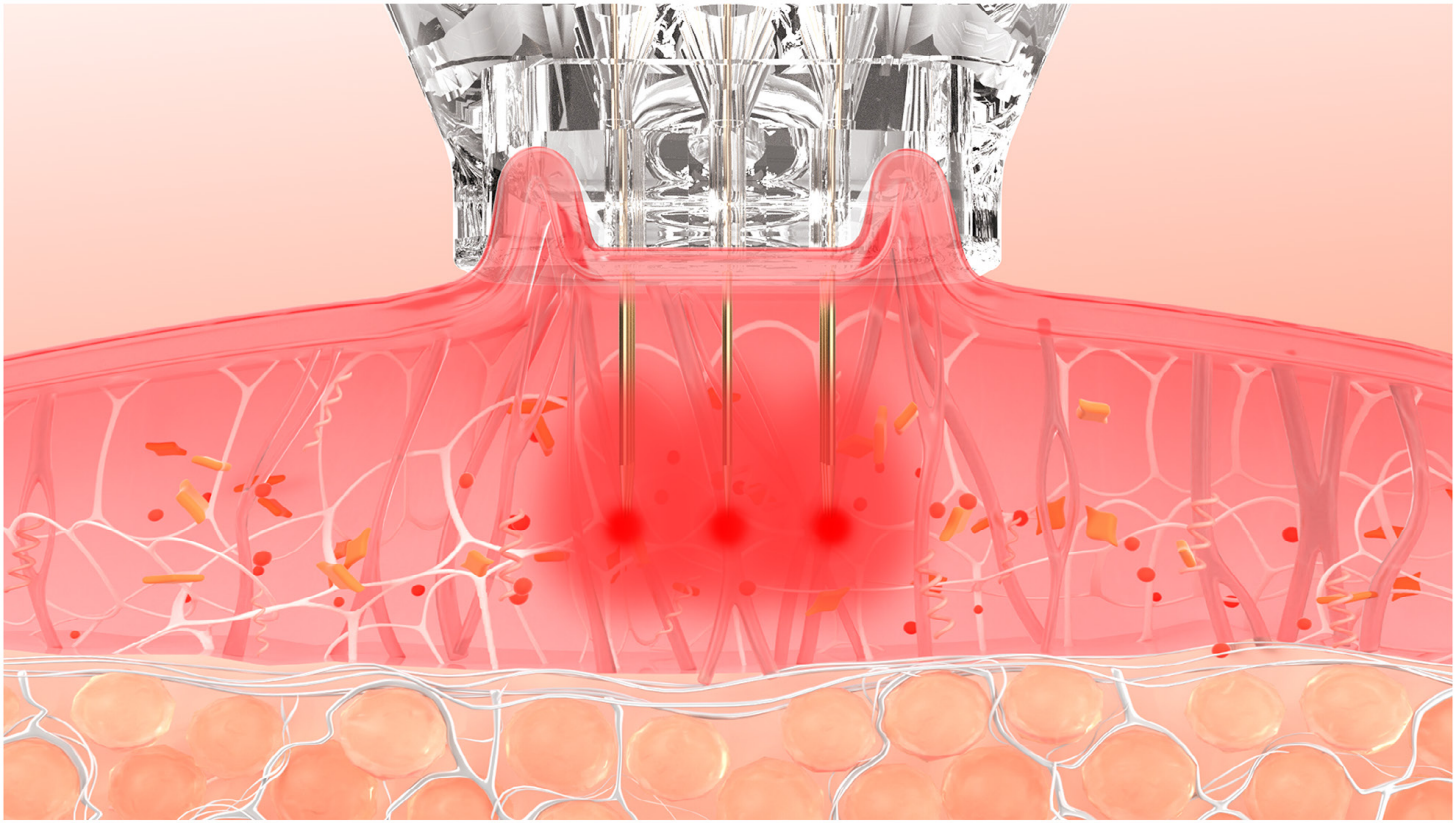
Schematic diagram of a novel vacuum‐assisted microneedle fractional radiofrequency system.

## 2 MATERIALS AND METHODS

We conducted this clinical, prospective, randomized, split‐face study to evaluate the efficacy of the novel vacuum‐assisted MFRS for patients with facial tightening. This study was approved by the Ethics Review Board of our hospital. All patients voluntarily signed the informed consent form before participating in the study (clinical trial registration number: ChiCTR 1900026090).

The study was performed at the Department of Laser and Aesthetic Medicine of our hospital for 12 months (January 2020–2021). Healthy individuals aged 25–65 years with facial skin laxity participated in the study. Patients were excluded if they were undergoing concomitant treatment of the facial skin, or had a history of botulinum toxin injection or insertion of soft tissue augmentation materials, or had undergone any skin rejuvenation treatment during the past 6 months, or a history of keloid formation, or active systemic or local infections, or were pregnant. Participants were randomized by independent statisticians. Based on the randomized enrollment results, half of the face underwent MFRS treatment and the other half was untreated (control). All participants were treated three times at intervals of 1 month. They were evaluated at 1, 3, and 6 months after the final treatment (Figure 2).

**FIGURE 2:**
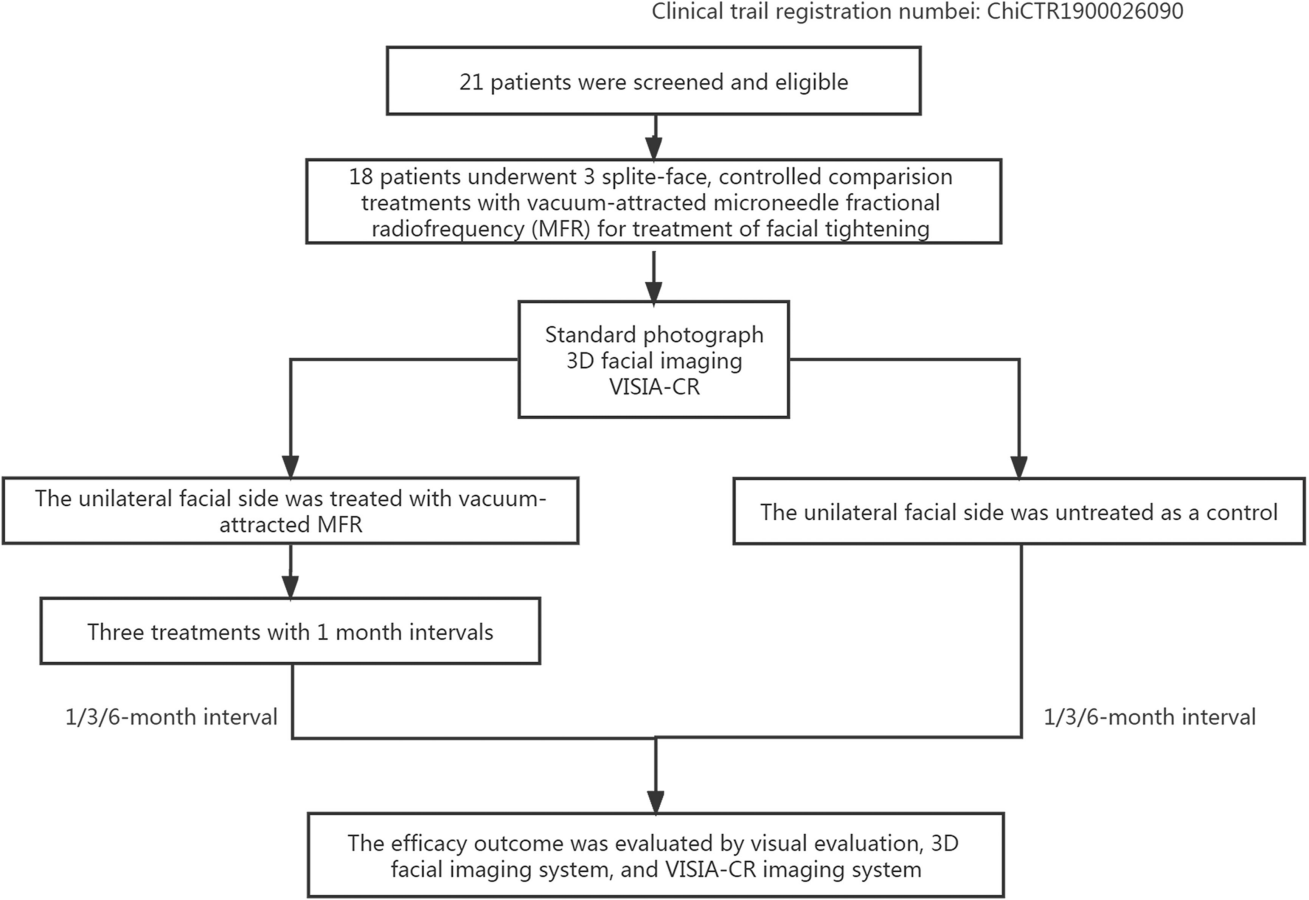
Patient enrollment and treatment randomization.

### 2.1 Treatment equipment and parameters

This study used the vacuum‐assisted MFRS (Gold Microneedle United II; Peninsula, Shenzhen, China). The treatment tip of the device comprises 5 × 5 microneedle electrode pairs per 1‐cm^2^ area. Each needle consists of two parts: a 0.3‐mm non‐insulated tip that generates radiofrequency energy and an insulated needle body with an adjustable length that protects the epidermis from thermal damage. The needle length can be freely adjusted from 0.5 to 3.0 mm, which allowing the generation of thermal coagulation to the desired layer.

### 2.2 Ex vivo study on porcine tissues

Simulations were performed on ex vivo porcine tissue to estimate skin and subcutaneous thermal effects and help to determine the proper treatment settings. Infrared thermal imaging and temperature under different conditions, with two power levels (6 and 12 W), and three pulse widths (100, 300, and 600 ms) were recorded using a thermal infrared imager (FLIR E95; FLIR System Inc., USA). After five passes of each setting, a setting of 6 W and 600 ms pulse width produced moderate heat to porcine tissue was selected (Table 1).

**TABLE 1:** Ex vivo infrared temperature on porcine tissue of the vacuum‐assisted MFRS.

Group	Power (W)	Pulse width (ms)	Depth of needle insertion	Pass	Initial temperature	End‐point temperature
1	6	100	3	5	22.0	26.2
2	6	300	3	5	22.6	29.4
3	6	600	3	5	22.8	31.4
4	12	100	3	5	23.5	28.6
5	12	300	3	5	23.8	32.1
6	12	600	3	5	24.8	34.4

### 2.3 Treatment process

The treatment parameters were as follows: vacuum‐assisted mode, power level of 6 W, and pulse duration of 600 ms. The microneedle depth was adjusted from 1.5 to 3 mm based on the relative skin thickness index and skin thickness measured by high‐resolution ultrasound.^6^, ^7^ The injection depths were as follows: 1.5 mm for the frontal and periorbital area (upper eyelid untreated), 2 mm for the zygomatic and cheek area, and 3 mm for the chin. The overlap rate was 75%. Superficial topical anesthesia with a mixture of 2.5% lidocaine hydrochloric acid and 2.5% prilocaine (Beijing Ziguang Medication Manufacture Corporation Ltd., Beijing, China) was applied to the cleansed treatment area 1 h before treatment, followed by disinfection with 70% alcohol. Negative‐pressure technology was adopted during treatment. When the skin was completely attracted by negative pressure from the tip, microneedles automatically penetrated to the set depth and emitted radiofrequency energy.

All patients underwent three treatment sessions at 1‐month intervals. Each session was conducted in an identical manner and in accordance with the aforementioned protocol. Identical treatment techniques were performed for all patients by two physicians. After the intervention, the treated areas were cooled with ice packs (cool mask/cool gel) for 20 min. All participants were instructed to gently cleanse their faces with tap water and avoid sun exposure.

### 2.4 Follow‐up and evaluation

Standardized clinical photographs of all patients were obtained using identical camera settings, lighting, and patient positioning before each treatment and at follow‐up (Canon 70D; Canon, Tokyo, Japan). Facial tightening efficacy was scored by three plastic surgeons and/or dermatologists not involved with this study using a blinded visual analog scale (VAS) with a score range of 0–10 (0 = worst; 1–2 = no improvement; 3–4 = slight improvement of 1%–25%; 5–6 = good improvement of 26%–50%; 7–8 = very good improvement of 51%–75%; and 9–10 = excellent improvement of 76%–100%). The scores were averaged if any discrepancy existed among the evaluators.

Patient faces were evaluated before each treatment and 1, 3, and 6 months after the final treatment using a three‐dimensional (3D) camera (3D LifeViz facial stereo system; QuantifiCare, Valbonne, France), and the volumes of the lower eyelids, cheek, and mandible were measured and recorded. The pretreatment and posttreatment 3D imaging could be automatically superimposed through the 3D imaging system, which helped to evaluate the volumetric changes objectively.^8^, ^9^, ^10^

Standard photographs of the patients were obtained. Wrinkles, spots, pores, ultraviolet spots, brown spots, red areas, and rhodopsin values were also recorded using VISIA‐CR (Canfield Scientific Inc., Fairfield, NJ). All the evaluation were conducted pretreatment and at 1‐, 3‐, and 6‐month follow‐up.

Participants rated their pain using the VAS pain score, which ranged from 0 (painless) to 10 (unbearable pain). Satisfaction with treatment was also rated by the patients and recorded using an anonymous questionnaire at 6 months after treatment (0 = very unsatisfactory; 1 = unsatisfactory; 2 = general; 3 = relatively satisfactory; and 4 = very satisfactory).

The posttreatment responses of each patient, including erythema, edema, hyperpigmentation, hypopigmentation, itchy skin, red rash, and any other side effects, were also recorded.

### 2.5 Data analysis

All data were analyzed using SPSS 20.0 (IBM, Armonk, NY) and GraphPad Prism 7 (GraphPad Software, Inc., San Diego, CA) to evaluate the effectiveness. The continuity variables are described as the mean ± standard deviation, and the stratified variables are described as the median ± quartile. For group comparisons, the paired *t* test was performed for normally distributed continuous variables, and the Wilcoxon paired rank sum test was used for the non‐normally distributed continuous variables. The level of significance was set at *p* < 0.05.

## 3 RESULTS

A total of 21 patients were included in this study, 18 patients (17 women and 1 man) completed all the treatments and follow‐up evaluations. Three patients were lost to follow‐up due to Covid. The age range was 30–63 years (mean age, 47.6 ± 9.74 years). Fifteen patients (83.3%) had Fitzpatrick Type III skin and three (16.7%) had Type IV skin.

Each patient was treated a total of three times, with 1‐month intervals between treatments. The differences in the VAS scores of the treated sides and control sides were 4.51 ± 1.38, 5.87 ± 1.28, and 5.80 ± 1.09 at 1, 3, and 6 months, respectively; these values were statistically significant (*p* < 0.05). The pairwise comparison of the VAS scores at 1, 3, and 6 months after treatment revealed that efficacy at 3 and 6 months was significantly better than that at 1 month after treatment (*p* < 0.05 and *p* < 0.05, respectively) and remained stable at 3 and 6 months (*p* > 0.05) after treatment (Figure 3A).

**FIGURE 3:**
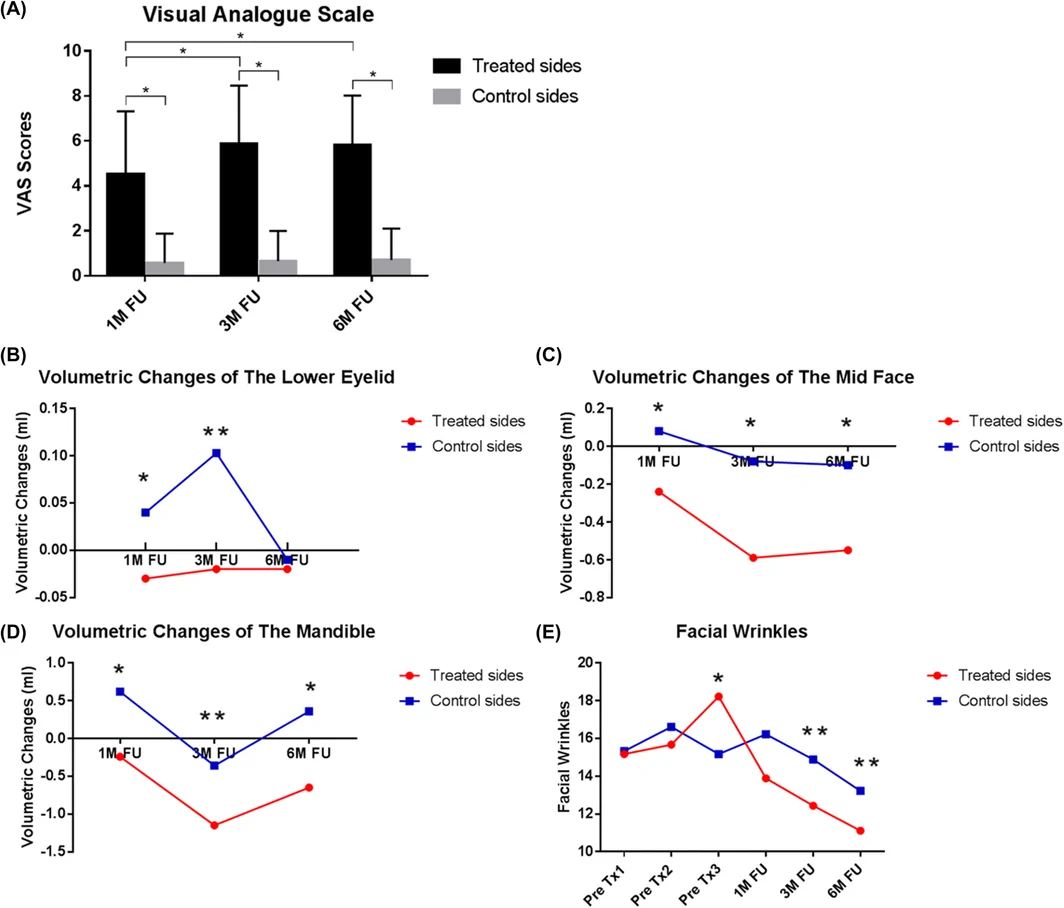
(A–E) (A), Comparisons of the visual analog scale (VAS) scores; (B–E), volumetric changes of the lower eyelid, midface, and mandible; changes in the number of facial wrinkles on the treated and control sides of each patient before treatment and 1, 3, and 6 months after treatment.

The volume changes according to the 3D camera for the treated lower eyelid side were −0.03 ± 0.12, −0.02 ± 0.13, and −0.02 ± 0.19 mL at 1, 3, and 6 months, respectively (positive values represent a volume increase; negative values represent a volume reduction); the changes were statistically significant compared to the control side (0.04 ± 0.15, 0.10 ± 0.53, −0.01 ± 0.16 mL, respectively; *p* < 0.05 for 1 and 3 months) (Figure 3B). The volume changes of the treated cheek at 1, 3, and 6 months were −0.24 ± 0.75, −0.59 ± 0.92, and −0.55 ± 0.65 mL; the changes were statistically significant compared to the control side (0.08 ± 0.70, −0.08 ± 0.53, and −0.10 ± 0.86 mL, respectively; *p* < 0.05) (Figure 3C). The volume changes of the treated mandible at 1, 3, and 6 months were − 0.24 ± 1.05, −1.15 ± 1.98, and − 0.65 ± 1.00 mL; the changes were also statistically significant (Figure 3D) compared to those of the control side (0.62 ± 1.26, −0.36 ± 2.11, and 0.36 ± 1.67 mL, respectively; *p* < 0.05).

An analysis using VISIA showed that the numbers of facial wrinkles on the treated and control sides were 15.17 ± 4.79 and 15.33 ± 4.83, respectively, before treatment (*p* > 0.05); these numbers were statistically significantly reduced to 12.44 ± 4.85 and 14.89 ± 5.26, respectively, at 3 months (*p* < 0.05). Furthermore, the efficacy sustained at 6 months, with wrinkle numbers of 11.11 ± 4.10 and 13.22 ± 4.44 for the treated and control sides, respectively (*p* < 0.05) (Figure 3E). No statistically significant changes in spots, pores, ultraviolet spots, brown spots, red areas, and rhodopsin values were detected (*p* > 0.05).

The mean score for pain sensation during treatment was 3.05 (standard deviation, 1.08). Five patients (27.78%) were very satisfied and 12 patients (66.67%) were relatively satisfied (Table 2). Among them, 13 subjects (72.22%) considered their skin tightness to be improved or significantly improved, and 14 (77.78%) considered their facial fine lines to be improved or significantly improved.

**TABLE 2:** Patient satisfaction scale and adverse events.

Likert Satisfaction Scale	No. of patients (%)
1 = Very dissatisfied	0 (0)
2 = Dissatisfied	0 (0)
3 = Slightly satisfied	1 (5.555)
4 = Satisfied	12 (66.67)
5 = Very satisfied	5 (27.78)
Adverse events	No. of patients (%)
Mild erythema	18 (100)
Scab	0 (0)
Edema	0 (0)
Blistering	0 (0)
Acne iformmiliaris	0 (0)
Hyperpigmentation	0 (0)
Hypopigmentation	0 (0)
Scarring	0 (0)

All patients (100%) developed mild erythema and purpura after treatment. They also reported a mild heat sensation after treatment. No patients developed scabs, itchy skin, red rash, hyperpigmentation, hypopigmentation, scars, or any other adverse events (Table 2).

Figures 4, 5, 6 show typical postoperative wrinkle improvement and facial tightening effects.

**FIGURE 4:**
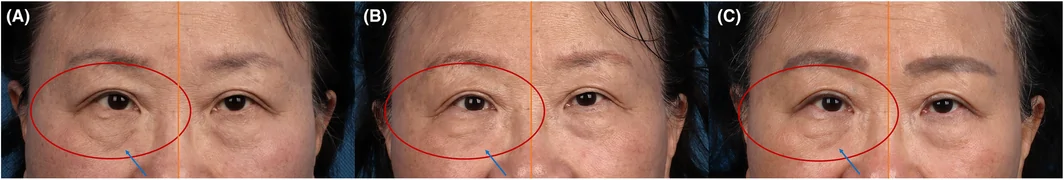
(A–C) A 62‐year‐old woman before treatment and 3 and 6 months after the last treatment. A significant improvement in the right‐side eye bags was observed. Before treatment (A), 3 months after the last treatment (B), and 6 months after the last treatment (C).

**FIGURE 5:**
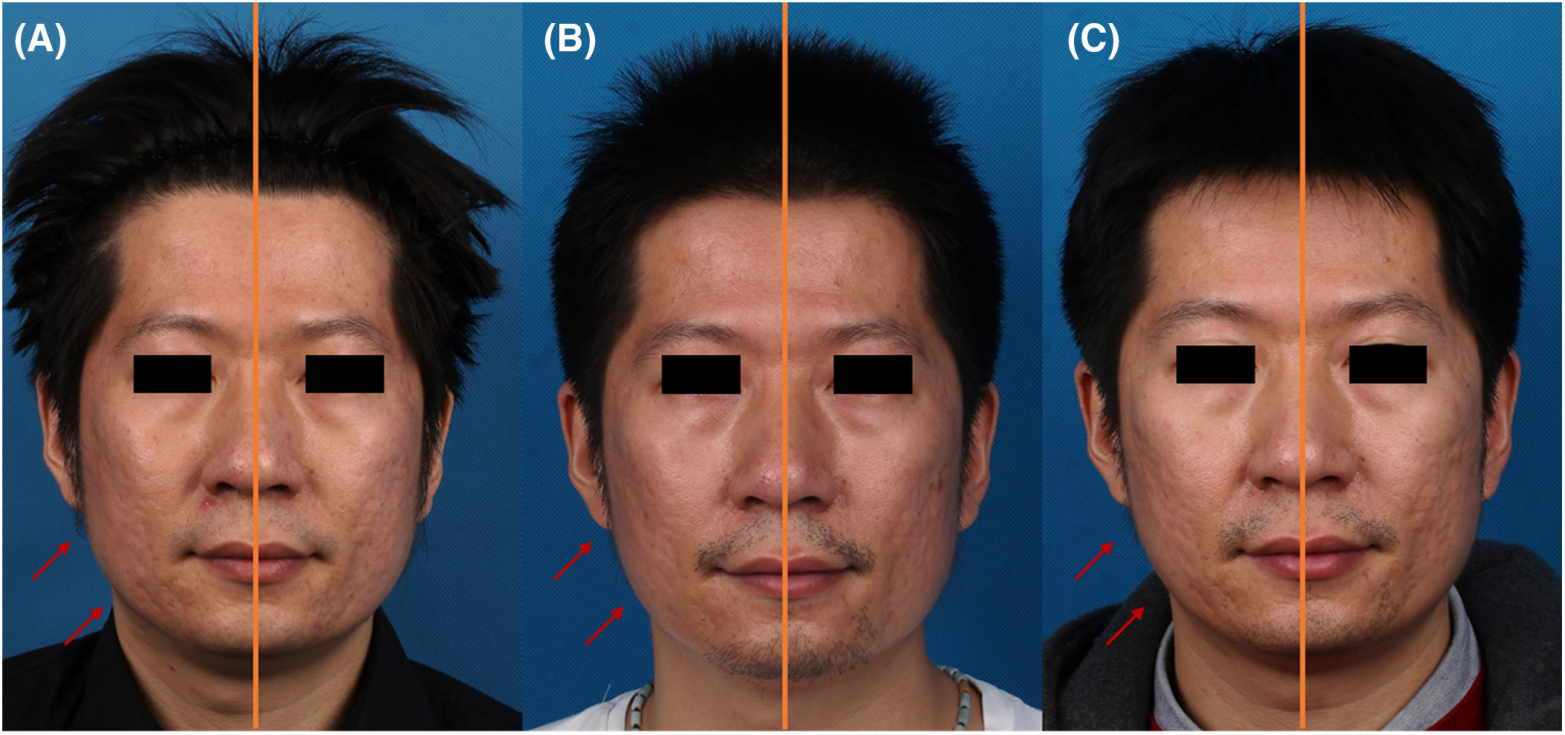
(A–C) A 35‐year‐old man before treatment (A) and 3 (B) and 6 (C) months after the last treatment. A significant improvement was observed. The contour of the mandible was significantly tightened on the right side of the face (Treated side).

**FIGURE 6:**
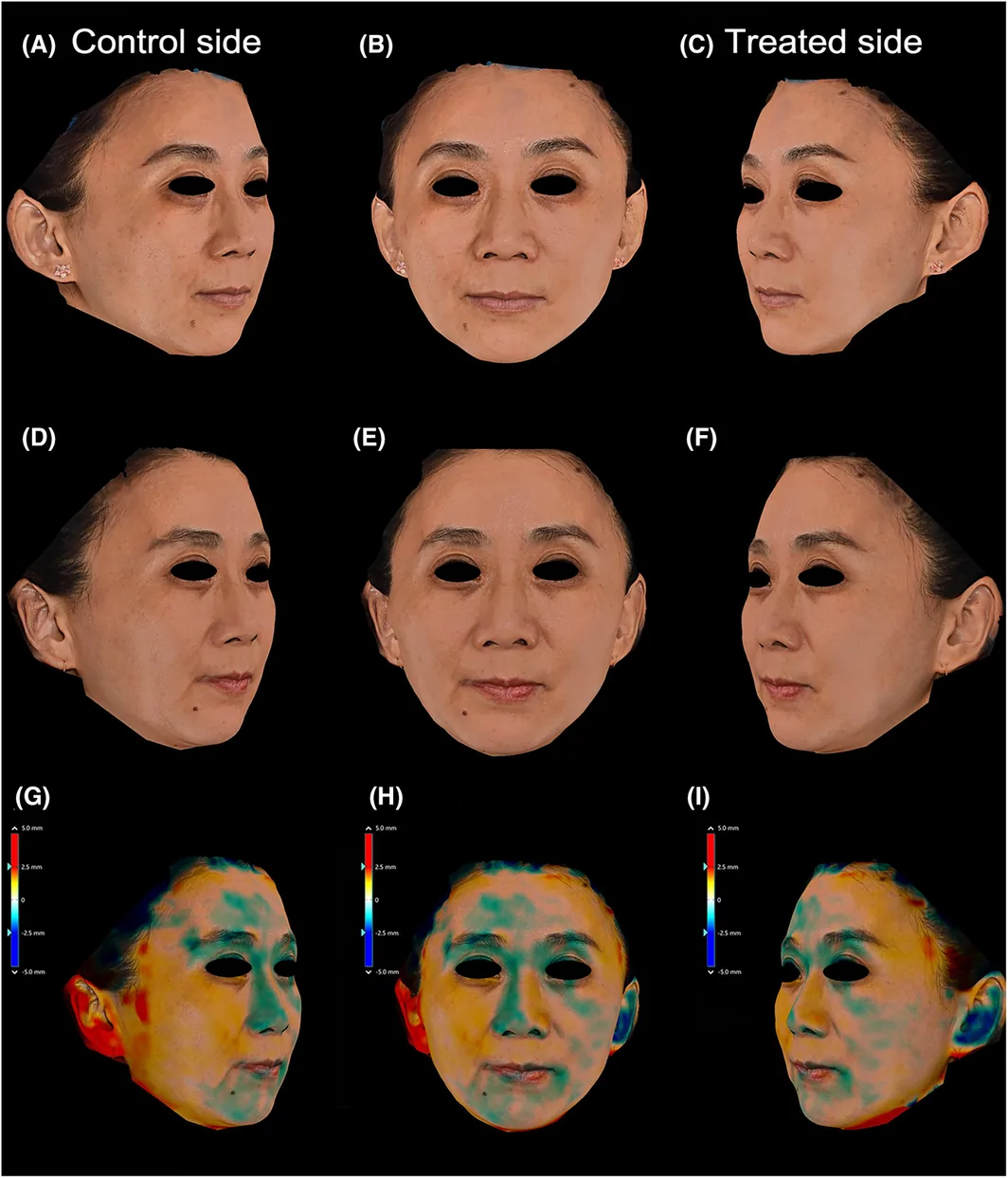
(A–I) A three‐dimensional (3D) imaging system was used to monitor facial volume changes. This figure presents a 40‐year‐old female subject's before‐treatment (A–C) and one‐month posttreatment (D–F). A superimposed 3D volumetric assessment is provided to compare the values before and 1 month after treatment (G–I). In the 2D color digital photographs posttreatment, the forehead, cheek, and mandibular regions appear tightened on the treated side (left face) when compared to the pretreatment condition. The extent of tightening is depicted using a color gradient, transitioning from light blue to dark blue to represent the increased degrees of achieved tightening.

## 4 DISCUSSION

Recently, the MFRS has become very popular among patients because of its safety, few side effects, and short recovery period. The insulated MFRS, with its energy concentrated on the tip, can significantly reduce the possibility of epidermal involvement and minimize the risks of epidermal burning, blistering, and pigmentation. The pinhole caused by the microneedle usually self‐heals within 24 h after the procedure.^11^ The MFRS is able to heat and coagulate the subcutaneous tissue including dermis, fascia and fat layer, depending on the depth it inserted, which inducing collagen regeneration, fascia contraction and fat lipolysis. Skin rejuvenation effects and tightened facial contour can be expected through such procedures.^12^, ^13^ Garg et al. evaluated the volume changes of four patients using Vectra‐H1 (Canfield Scientific Inc.) and ultrasound imaging and verified that the radiofrequency targets facial collagen as deep as the facial fat, thereby achieving “reversal” of the grooves and bulges of aging with volume redistribution and favorable “remolding” of fat pads.^14^ Tanaka et al. also performed monthly 3‐D volumetric assessments for 6 months after microneedle radiofrequency treatment, indicating significant median volumetric reduction posttreatment and providing safe and effective treatment to skin tightening in Asian patients.^15^

The traditional MFRS procedure relies on the hand strength to overcome skin resistance and reach the desired skin depth. Inadequate penetration may lead to epidermal burn as well as increase uncertainty of the tightening efficacy. Furthermore, sideling instead of vertical insertion into the skin of the microneedle tip is another wrong but hard to be evitable operation for inexperienced clinician leading to undesirable side effects.^16^ Therefore, a new‐generation MFRS with negative‐pressure technology, which can automatically attract the skin with negative pressure and detect adsorption intelligently, was introduced to help energy emission more accurately and safely. Since the face contour is uneven, the vacuum‐assisted technology is more able to help the microneedle tip to reach the preset depth and increase the treatment accuracy. The technique also helps the MFRS to be easier for clinician to command.

The negative pressure also can attract and raise the skin before emitting heat, thus avoiding the possible burn risks of subcutaneous tissue such as eyebrow. With this negative‐pressure technology, the periorbital area can be treated safely, even without placing the eye shield. A deeper insertion depth can be achieved safely to tighten the periorbital region (the needle depth can reach up to 1.3–1.5 mm when treating the periorbital area (upper eyelid untreated)). The insertion depth on the mandible region can accurately reach to 3 mm with vacuum‐assisted technique, which is deep enough to induce lipolysis effects in the superficial fat layer and contract superficial muscular aponeurotic system (SMAS), leading to the tightening and lifting of mandibular contour.^4^, ^17^, ^18^

The VAS pain score was recorded to be relatively low, comparing to the other non‐vacuum‐assisted MFRS.^17^, ^19^, ^20^ We believe the negative pressure on the skin may distract subjects' attention and help to reduce pain sensation in the emission process.

A reduction of facial wrinkles was detected using VISIA, however, the number of the enlarged facial pores did not differ significantly posttreatment, which is inconsistent with the results reported previously. This difference may contribute to the deeper insertion depth adopted in the study. On the other side, the changes of red areas and brown spots were not significant, which indicated safety for the device to treat subjects with melasma and dark skin.^21^

Limitations include small sample size and relatively short follow‐up period. Studies with larger sample sizes and longer follow‐up periods should be performed to confirm our results and further observe the long‐term effect of the novel MFRS. Clinical scales should be integrated into future studies to enhance the credibility and objectivity of the research. Additionally, B‐ultrasonography and histological examination will be employed to measure the depth of the skin and subcutaneous tissue.

## 5 CONCLUSION

This study is the first prospective, randomized study to evaluate the novel vacuum‐assisted MFRS for the treatment of facial photoaging. The vacuum‐assisted MFRS is safe and effective and should be recommended to improve fat‐pad sagging and reduce wrinkles. The effects of the MFRS are sustained for at least 6 months. Furthermore, the vacuum‐assisted technology increases the accuracy and safety to the treatment.

## AUTHOR CONTRIBUTIONS

Xiaoxi Lin and Yue Han had full access to all of the data in the study and takes responsibility for the integrity of the data and the accuracy of the data analysis. *Study concept and design*: Xiaoxi Lin, Yue Han, Wenxin Yu. *Acquisition, analysis, or interpretation of data*: Ying Liu, Wenxin Yu, Yue Han, Qingqing Cen, Lucia Zhou. *Drafting of the manuscript*: Yue Han, Wenxin Yu, Jiafang Zhu. *Critical revision of the manuscript for important intellectual content*: Xiaoxi Lin, Ying Liu, Jiafang Zhu. *Statistical analysis*: Ying Shang. Obtained funding: Xiaoxi Lin, Yue Han. *Administrative, technical, or material support*: Wenxin Yu, Jiafang Zhu, Ying Liu. *Study supervision*: Xiaoxi Lin, Gang Ma.

## FUNDING INFORMATION

This study was supported by grants from the National Natural Science Foundation of China (No.81971847, No.81772100, and No.81571917), Clinical Research Project of Shanghai Ninth People's Hospital, Shanghai Jiao Tong University School of Medicine (No. JYHX2019003, and No.JYHX2022010), project ZR2021QH291 supported by Shandong Provincial Natural Science Foundation, and Clinical Research Project of Nanfang Hospital, Southern Medical University (No. 2023CR033).

## ETHICS STATEMENT

This study was approved by the Ethics Review Board of Shanghai Ninth People’s Hospital, Shanghai Jiao Tong University School of Medicine. Approval number: SH9H‐2019‐T127‐2.

## CLINICAL TRIAL REGISTRATION INFORMATION

ChiCTR1900026090.

## Data Availability

The data that support the findings of this study are available on request from the corresponding author. The data are not publicly available due to privacy or ethical restrictions.
